# RECURRENT FALLS IN OLDER ADULTS ARE ASSOCIATED WITH MORTALITY AND HOSPITALIZATION

**DOI:** 10.1093/geroni/igad104.3558

**Published:** 2023-12-21

**Authors:** Samita Heslin, Elinor Schoenfeld, Melanie Keister, Sritha Rajupet, Kristi Ladowski, Adam Singer

**Affiliations:** Stony Brook University, Stony Brook, New York, United States; Stony Brook University School of Medicine, Stony Brook, New York, United States; LINCATS – Associate Director of Technology and Resources, Stony Brook, New York, United States; Stony Brook University, Stony Brook, New York, United States; Stony Brook University, Stony Brook, New York, United States; Stony Brook University, Stony Brook, New York, United States

## Abstract

Annually, 1 in 4 older adults fall and roughly half have a second fall within 6 months. We studied 57 hospitals in the TriNetX Network. We included all older adults discharged home from the emergency department (ED) after a fall in 2022 for a total of 89,061 patients. Of these, 13,389 (15%) patients returned to the ED for another fall within 6 months. Patients with repeat falls were older (78.7 vs 76.5 years), more likely to have hypertension (76 vs 56%), prior myocardial infarction (14 vs 3%), heart failure (29 vs. 15%), peripheral vascular disease (18 vs 10%), cerebrovascular event (19 vs 10%), chronic obstructive pulmonary disease (COPD) (23 vs 14%), diabetes (40 vs 23%), liver disease (17 vs 11%), chronic kidney disease (30 vs 16%), and cancer (45 vs 32%); P< 0.001. Patients with recurrent falls were more likely to be prescribed cardiovascular medications (84 vs. 66%), central nervous system medications (87 vs 69%), opioids (71 vs 52%), sedatives/hypnotics (59 vs 42%), antipsychotics (23 vs 12%), antidepressants (51 vs 32%), antiparkinsonian agents (9 vs 4%), and antivertigo agents (15 vs 9%); P< 0.001. Patients with a recurrent fall within 6 months had higher hospital admission rate (46.2 vs 15.8%, OR 4.6 [95%CI, 4.4-4.7]) and higher mortality rate (6.2 vs 3.5%, OR 1.83 [95%CI 1.7-2.0]). Older adults discharged home from the ED after falling are at high risk of a second fall and those who have a repeat fall within 6 months are at higher risk of hospital admission and mortality.

